# Distinctive sphingolipid patterns in chronic multiple sclerosis lesions

**DOI:** 10.1194/jlr.RA120001022

**Published:** 2020-08-07

**Authors:** Maria Podbielska, Zdzislaw M. Szulc, Toshio Ariga, Anna Pokryszko-Dragan, Wojciech Fortuna, Małgorzata Bilinska, Ryszard Podemski, Ewa Jaskiewicz, Ewa Kurowska, Robert K. Yu, Edward L. Hogan

**Affiliations:** 1 Department of Biochemistry and Molecular Biology, Medical University of South Carolina, Charleston, SC, USA; 2 Laboratory of Microbiome Immunobiology, Ludwik Hirszfeld Institute of Immunology and Experimental Therapy, Polish Academy of Sciences, Wroclaw, Poland; 3 Department of Neuroscience and Regenerative Medicine, Augusta University, Medical College of Georgia, Augusta, GA 30912, USA; 4 Department of Neurology, Wroclaw Medical University, Wroclaw, Poland; 5 Department of Neurosurgery, Wroclaw Medical University, Wroclaw, Poland; 6 Bacteriophage Laboratory, Ludwik Hirszfeld Institute of Immunology and Experimental Therapy, Polish Academy of Sciences, Wroclaw, Poland; 7 Laboratory of Glycobiology, Ludwik Hirszfeld Institute of Immunology and Experimental Therapy, Polish Academy of Sciences, Wroclaw, Poland; 8 Department of Neurology, Medical University of South Carolina, Charleston, SC, USA

**Keywords:** brain lipids, central nervous system, ceramides, ceramide 1-phosphate, clinical lipidology, inflammation, lipidomics, neurodegeneration, neurological diseases, mass spectrometry

## Abstract

Multiple sclerosis (MS) is a CNS disease characterized by immune-mediated demyelination and progressive axonal loss. MS-related CNS damage and its clinical course have two main phases: active and inactive/progressive. Reliable biomarkers are being sought to allow identification of MS pathomechanisms and prediction of its course. The purpose of this study was to identify sphingolipid (SL) species as candidate biomarkers of inflammatory and neurodegenerative processes underlying MS pathology. We performed sphingolipidomic analysis by HPLC-tandem mass spectrometry to determine the lipid profiles in post mortem specimens from the normal-appearing white matter (NAWM) of the normal CNS (nCNS) from subjects with chronic MS (active and inactive lesions) as well as from patients with other neurological diseases. Distinctive SL modification patterns occurred in specimens from MS patients with chronic inactive plaques with respect to NAWM from the nCNS and active MS (Ac-MS) lesions. Chronic inactive MS (In-MS) lesions were characterized by decreased levels of dihydroceramide (dhCer), ceramide (Cer), and SM subspecies, whereas levels of hexosylceramide and Cer 1-phosphate (C1P) subspecies were significantly increased in comparison to NAWM of the nCNS as well as Ac-MS plaques. In contrast, Ac-MS lesions were characterized by a significant increase of major dhCer subspecies in comparison to NAWM of the nCNS. These results suggest the existence of different SL metabolic pathways in the active versus inactive phase within progressive stages of MS. Moreover, they suggest that C1P could be a new biomarker of the In-MS progressive phase, and its detection may help to develop future prognostic and therapeutic strategies for the disease.

Multiple sclerosis (MS) is a polyphasic immune-mediated disorder characterized by multifocal inflammatory infiltrates (T cells, B cells, and macrophages) within the CNS, with concomitant degradation of the myelin sheath, oligodendrocytes, and axons, along with reactive astrogliosis and activated microglia (1).

Multiple areas of myelin loss within the CNS called “plaques” or “lesions” are the pathologic hallmark of MS. It is evident that MS lesions evolve differently during the early/acute versus the chronic phase of the disease, and within each phase, different plaque types occur in particular stages of activity (2). Furthermore, it is well-known that degradation of minor myelin proteins (myelin oligodendrocyte glycoprotein, myelin-associated glycoprotein, 2′,3′-cyclic-nucleotide 3′-phosphodiesterase) denotes early active plaques, whereas the presence of major myelin proteins (proteolipid protein, myelin basic protein) indicates late active lesions. Inactive lesions are infiltrated by macrophages that lack myelin debris but may still contain empty vacuoles or periodic acid-Schiff-positive degradation products, the results of the macrophages’ inability to digest the myelin neutral lipid components (2). As the plaque progresses from acute/active to chronic/inactive, its edema resolves, inflammation decreases, and macrophages and microglia gradually disappear. Astrocytes produce glial scars that fill the demyelinated plaque. These characteristics prompted Charcot to name these lesions as sclerotic plaques (3), appearing as a major autopsy finding in MS subjects.

MS-related damage to CNS tissues has been found to include two main pathological processes: inflammatory myelin destruction (demyelination) and progressive irreversible axonal loss (neurodegeneration). The underlying pathology of the inflammatory component is generally believed to be associated with an autoimmune attack upon myelin antigens. However, extensive studies have not yet established the predominant target antigenic structures involved in the autoimmune response most relevant for MS background (4). Both processes were shown to be initiated at the disease onset, but they develop with different dynamics: the peak of inflammatory activity occurs in the early stages of MS, while neurodegeneration with axonal loss is gradually escalating toward more advanced progressive stages (5). Contribution of these processes to MS-related CNS damage corresponds with the clinical course of MS, defined as relapsing-remitting, secondary progressive, or primary progressive. A more recent concept of MS course assumes distinguishing two main phases of the disease: active and inactive/progressive, which may be temporarily overlapping (6). Despite significant progress in diagnostics and therapeutic advances in recent years, there are still some problems that need to be elucidated. First, great individual variability of MS course and response to treatment hinders the prognosis of the disease outcome. Another challenge is associated with managing the inactive/progressive phase of the disease, while available treatment options almost exclusively target the active one. Therefore, reliable biomarkers are being sought to allow identification of the disease pathogenic mechanisms and prediction of its clinical course. There is sufficient evidence for relevant indices of inflammatory activity [intrathecal IgG synthesis, level of cytokines and chemokines or adhesion molecules in cerebrospinal fluid (CSF)] as well as neurodegeneration (level of neurofilaments and chitinase in CSF) in the CNS. However, none of these markers turned out to be specific for MS, which limits their diagnostic and predictive value. Thus, there is a need to investigate new and more relevant biomarkers potentially useful in MS (7, 8).

Sphingolipids (SLs), as the major component of CNS myelin sheaths, seem to be potential biomarker candidates in MS (9). They participate in numerous inflammatory processes and are responsible for controlling intracellular trafficking and signaling, cell growth, adhesion, vascularization, survival, and apoptosis (10–12). Although SL-specific antibodies and T cells have been identified in MS (13, 14), very little is known about lipid composition in particular stages of MS plaques (acute vs. chronic) (15) as well as the role of bioactive lipids in CNS autoimmunity. SLs also exert pronounced effects on inflammation, in the context of autoimmunity, by acting either as targets or regulators of the immune response. In addition, myelin sheath lipids have been reported to induce apoptosis in autoreactive T cells (16) and ameliorate experimental autoimmune encephalomyelitis (17, 18). In particular, ceramide (Cer) and the enzymes linked to its production have been described to play a pivotal role in oligodendrocyte damage and demyelination (18–22). It seems that lipids in the CNS induce perturbations in the balance of anti- and pro-inflammatory that are essentially involved in MS pathology. Recent evidence suggests that alterations in SL pathways may reflect disease activity (23, 24). Due to the activity of some essential hydrolytic enzymes, such as SMases, Cers of different chain lengths may participate in different cellular processes, such as differentiation, proliferation, and programmed cell death (12, 25).

Comprehensive profiling of CNS lipids in MS lesions might provide a better insight into their role in the pathogenesis of the disease, including an attempt to define the metabolic pathways leading to autoimmune demyelination and/or neurodegeneration. Such findings might contribute to evaluate the usefulness of SLs as biomarkers of various phases of MS or even as potential targets for therapeutic interventions. Myelin lipids in MS have already been investigated in some studies, but few of them analyzed their distribution in brain MS tissues (17, 26, 27) and in CSF (19, 28–30), which might allow a direct insight into disease-related CNS damage.

In this study, we performed a targeted sphingolipidomic analysis of post mortem brain tissues in patients with MS as well as subjects with the CNS affected by other neurological diseases (ONDs) or with substantially normal CNS (nCNS). The comparative analyses were further conducted for particular types of MS lesions, namely chronic active and chronic inactive. We have observed the distinctive pattern of SL metabolism in chronic inactive MS (In-MS) lesions in comparison to the nCNS. Based on our findings, we propose Cer 1-phosphate (C1P) to be a potential new biomarker of the MS progressive phase.

## MATERIALS AND METHODS

### Human autopsy brain tissues

Fresh frozen brain tissues were obtained from the Human Brain and Spinal Resource Center, Veterans Affairs West Los Angeles Healthcare Center, 11301 Wilshire Blvd., Los Angeles, CA 90073, which is sponsored by the National Institutes of Health, the National MS Society, and the US Department of Veterans Affairs. Brain tissue specimens were derived from autopsy of patients with clinically diagnosed and neuropathologically confirmed MS (n = 13) and ONDs (n = 15) as well as from three controls who had been diagnosed with diseases without CNS involvement, with essentially normal brain confirmed on autopsy findings, nCNS. OND samples were further subdivided into inflammatory OND (I-OND; n = 5) and noninflammatory OND (NI-OND; n = 10) reference subgroups. Patients’ clinical and autopsy characteristics are provided in **Table 1**. All procedures performed in this study were in accordance with ethical standards of the institutional ethics committees. Informed consent was obtained at the University of California Los Angeles from the human subjects or their representatives, and Declaration of Helsinki principles were followed. Preservation of anonymity and confidentiality and masking of samples were maintained throughout all studies.

**TABLE 1.** Patient clinical characteristic details

Sample Number	Brain Region	Tissue Character	Gender	Age	Post Mortem Interval (h)	Clinical Diagnosis	Group
4467	Frontal cortex*^a^*	Plaque	Female	62	23.0	MS	Ac-MS
4218	Frontal cortex*^a^*	Plaque	Female	63	15.0	MS	
4546	Frontal cortex*^a^*	Plaque	Male	59	38.5	MS	
4477	Frontal cortex*^a^*	Plaque	Male	67	19.0	MS	
4503	Frontal cortex*^a^*	Plaque	Female	54	24.0	MS	In-MS
3867	Frontal cortex*^a^*	Plaque	Male	75	13.0	MS	
4959	Frontal cortex*^b^*	Plaque	Female	47	22.1	MS	
4934	Frontal cortex*^b^*	Plaque	Female	51	17.5	MS	
5056	Frontal cortex*^b^*	Plaque	Female	59	20.1	MS	
4832	Frontal cortex*^b^*	Plaque	Male	54	23.0	MS	
4663	Frontal cortex*^b^*	Plaque	Male	62	16.1	MS	
5154	Frontal cortex*^b^*	Plaque	Male	63	13.0	MS	
5268	Frontal cortex*^b^*	Plaque	Male	66	17.0	MS	
4471	Frontal cortex*^a^*	NAWM	Female	73	12.0	Chronic encephalitis of Rasmussen	I-OND
4403	Frontal cortex*^b^*	NAWM	Female	77	18.3	Herpes simplex type I encephalitis	
747	Frontal cortex	NAWM	Female	66	26.0	Subacute-chronic encephalitis (HSV, HE, etc.) without inclusion	
1418	Frontal cortex	NAWM	Male	69	4.5	Chronic encephalitis, etiology unknown	
924	Frontal cortex	NAWM	Male	86	24.0	Herpes zoster encephalitis	
4222	Frontal cortex*^a^*	NAWM	Female	72	15.0	Parkinson’s disease	NI-OND
3780	Frontal cortex*^a^*	NAWM	Female	92	21.3	Parkinson’s disease	
3942	Frontal cortex*^a^*	NAWM	Male	81	15.0	Parkinson’s disease	
3746	Frontal cortex*^c^*	NAWM	Female	69	24.0	Parkinson’s disease	
3934	Frontal cortex*^c^*	NAWM	Female	88	19.3	Parkinson’s disease	
3761	Frontal cortex*^c^*	NAWM	Female	83	12.0	Multi-infarct dementia (clinical only)	
3769	Frontal cortex*^c^*	NAWM	Male	63	14.0	Parkinson’s disease	
3742	Frontal cortex*^c^*	NAWM	Male	74	23.0	Parkinson’s disease	
3643	Frontal cortex*^c^*	NAWM	Male	81	19.0	Parkinson’s disease	
3779	Frontal cortex	NAWM	Male	68	21.3	Dystonia	
5072	Frontal cortex*^a^*	NAWM	Male	83	19.5	Chronic obstructive pulmonary disease	nCNS
5190	Frontal cortex*^a^*	NAWM	Male	68	20.3	Heart attack	
3750	Frontal cortex*^b^*	NAWM	Male	77	12.3	Congestive heart failure	

a Subcallosal stratum close to lateral ventricle.

b Above caudate nucleus close to lateral ventricle.

c Radiation of corpus callosum.

### Histopathology and immunochemistry

Normal-appearing and pathological tissues were selected by gross examination and verified by microscopic examination. To assess the presence of myelin and identify areas of demyelinated plaques and normal-appearing white matter (NAWM), frozen 4 μm-thick cryostat tissues sections were stained with Luxol fast blue and H&E myelin stains. Neuropathological evaluation also comprised staining for axons (Bielschowsy’s silver impregnation). This allowed identification of lymphocytes and plasma cells as well as foamy macrophages containing myelin degradation products. The stage of lesional development was determined immunochemically as well. Anti-CD68 antibody was used to identify macrophages (data not shown). MS lesions were classified as either chronic active MS (Ac-MS) (profound inflammation with macrophages present) or In-MS (immunologically silent).

### Tissue homogenization and protein determination

Part of each frozen human brain tissue (100 mg) was cut and homogenized with 2 ml of tissue homogenization buffer containing 0.25 M sucrose, 25 mM KCl, 50 mM Tris-HCl, and 0.5 mM EDTA (pH 7.4). Homogenization was performed on ice using a Polytron electric homogenizer until no solid pieces were observed. Next, 100 μl of the tissue homogenate was diluted 1:10 with the homogenization buffer and aliquots of 10 μl were taken for protein determination assay using a Pierce BCA protein assay kit. Aliquots corresponding to 1 mg of protein were transferred to 15 ml Falcon tubes and subjected to lipid extraction.

### Lipid extraction

All solvents were analytical grade from Fisher Scientific (Hampton, NH). SL standards were from the Medical University of South Carolina Lipidomics Shared Resource or from a commercially available source (Avanti Polar Lipids, Matreya LLC) with purity of ≥98%.

Homogenates of tissues (1 mg per protein) were fortified with 50 μl of the appropriate internal standards (ISs), specifically: sphingosine (Sph)/Cer/dihydroceramide (dhCer) ISs [17C base D-*erythro*-sphingosine (17C/Sph), 17C base D-*erythro*-S1P (17C/S1P), 17C base D-*erythro*-dihydrosphingosine (17C/dhSph), D-*erythro*-N-palmitoyl-13C-D-*erythro*-sphingosine (13C/C16-Cer), N-heptadecanoyl-D-*erythr*o-sphingosine (18C/C17-Cer), N-heptadecanoyl-D-*erythr*o-dihydrosphingosine (18C/C17-dhCer), D-*erythro*-N-palmitoyl-17C-D-*erythro*-sphingosine (17C/C16-Cer), D-*erythro*-N-nervonoyl-17C-D-*erythro*-sphingosine (17C/C24:1-Cer)]; hexosylceramide (HexCer)/lactosylceramide (LacCer) ISs [18C/C8-glucosylceramide (GluCer), 18C/C12-GluCer, 18C/C8-LacCer, 18C/C12-LacCer]; C1P ISs [17C/C16-C1P, 17C/C18:1-C1P, 17C/C24-C1P]; and SM ISs [D-*erythro*-C6-SM (18C/C6-SM), D-*erythro*-C17-SM (18C/C17-SM)].

Lipids were extracted with 2 ml of one phase solvent system containing ethyl acetate/isopropanol/water (60/30/10%; v/v). The upper organic phase was transferred to a glass tube. To the remaining diluted tissue homogenates, an additional 2 ml of extraction solution was added to further facilitate complete extraction. The upper organic phase was then transferred and added to the glass tube containing the initial extract (total 4 ml extract). The lipid extract was divided into two parts. Part A (1 ml) was subjected to base mild alkaline hydrolysis in order to remove glycerolipids interfering with SM analysis, as reported previously (31), and used for analysis of SM subspecies. The remaining 3 ml of extract (part B) was used for analysis of Cer, dhCer, sphingoid bases and their 1-phosphate derivatives as well as HexCer, LacCer, and C1P. Both extracts, A and B, after evaporation and reconstitution in 150 μl of acidified (with 0.2% formic acid) methanol, were stored at 4°C prior to injection on the HPLC-tandem mass spectrometry system.

### Sphingolipidomic analysis by HPLC-tandem mass spectrometry

Analyses of SLs were performed by HPLC-tandem mass spectrometry at the Medical University of South Carolina Lipidomics Shared Resource. SLs analyzed included sphingoid bases (C18:1, C18:0); Sph and dhSph, their 1-phosphates [S1P and dihydrosphingosine 1-phosphate (dhS1P)] as well as dhCer, Cer, SM, HexCer, LacCer, and C1P species. HPLC-tandem mass spectrometry analysis was performed on a Thermo Fisher TSQ Quantum or SCIEX Q-Trap triple-stage quadrupole mass spectrometer, operating in a multiple reaction monitoring positive ionization mode, as previously described (32, 33). Chromatographic separations were obtained under a gradient elution using mobile phase A consisting of 2 mM ammonium formate in 0.2% formic acid in water and mobile phase B consisting of 1 mM ammonium formate in 0.2% formic acid in methanol on the BDS Hypersil C8, 150 × 3.2 mm, 3 μm particle size column.

Peaks corresponding to the target analytes of SLs and ISs were collected and processed using the Xcalibur™ software system (Fisher Scientific). Quantitative analysis was based on calibration curves using a linear regression model as described previously (20). SLs with unavailable standards were quantified using the calibration curve of their closest counterpart.

### Statistical analysis

SL levels for each sample were calculated by summing up the total number of all SL subspecies measured expressed in picomoles per milligram of protein and then normalizing that total to 100%. Because of the uneven distribution of our data, a nonparametric test was used for each two group comparison. Statistical differences between groups were determined by Mann-Whitney test using GraphPad PRISM 7.01, with *P* < 0.05 being considered as statistically significant.

## RESULTS

### Characteristics of MS lesions studied

**Fig. 1.**
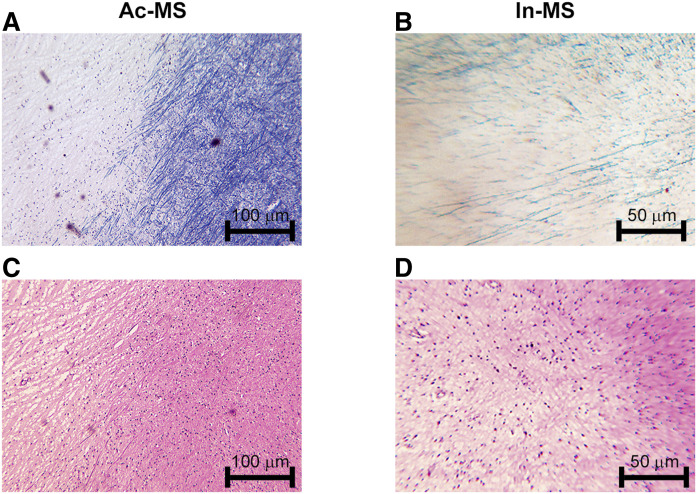
Plaque morphology in MS cases examined. Tissue sections were stained with Luxol fast blue (A, B) and H&E (C, D). Chronic Ac-MS plaque was characterized by loss of myelin in the left side (A) and visible disperse inflammatory lymphocyte infiltration (C). Chronic In-MS lesion indicated demyelinization in the bottom of the left corner (B) and no evidence of inflammation (D). Original magnification: 100× (A, C) and 200× (B, D); scale bars have been inserted in the micrographs.

Histopathological analysis in clinically diagnosed cases of MS revealed features typical for this disease such as demyelination, oligodendrocyte and axonal loss, inflammation with evidence of monocyte infiltrates present, and some degree of gliosis. MS lesions were classified depending on their activity, examples of which are shown in **Fig. 1**. Of the 13 plaques examined, 4 were classified as chronic active and 9 were categorized as chronic inactive type (Table 1).

### SL profile in NAWM of the unaffected CNS

**Fig. 2.**
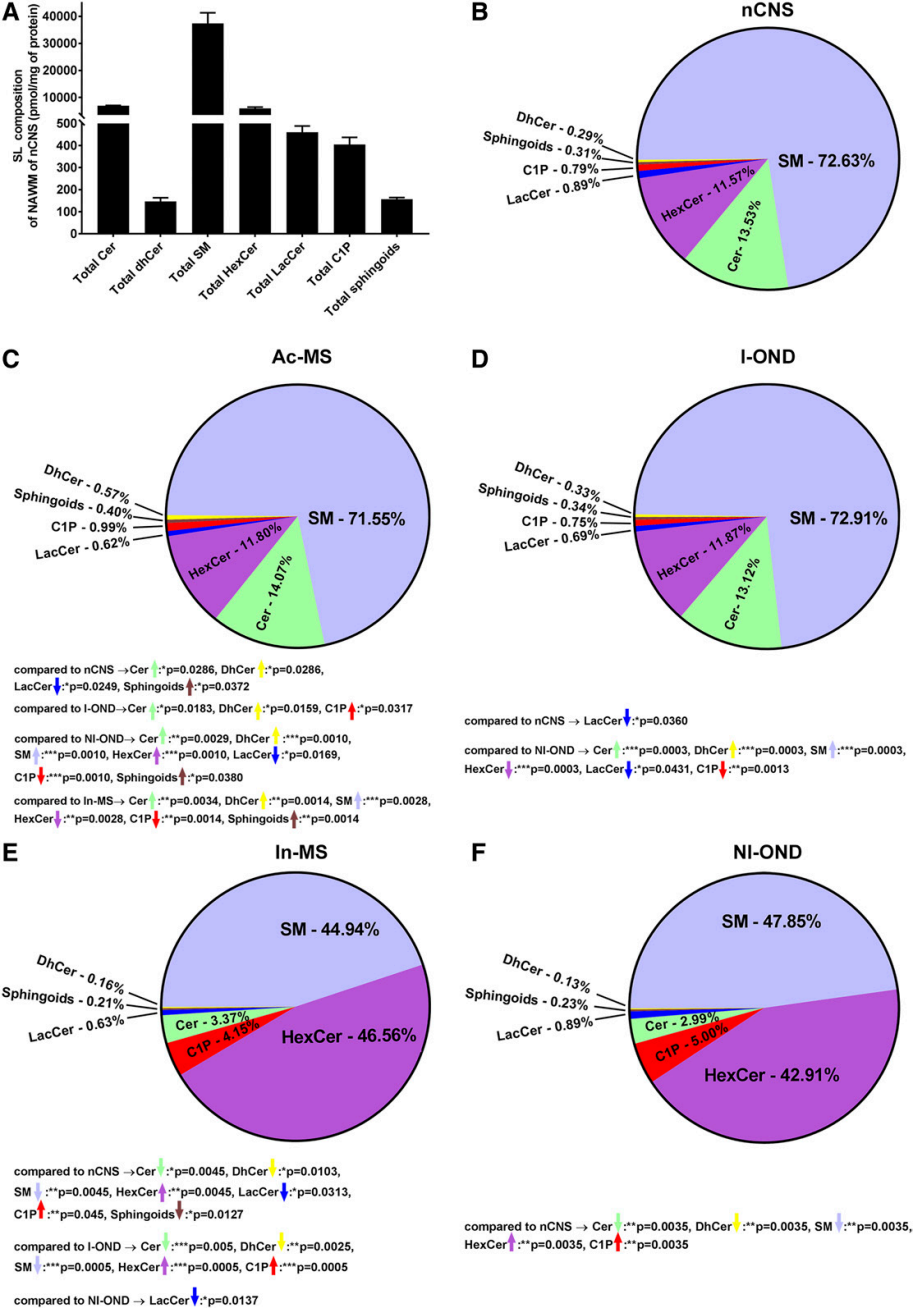
SL profiles in post mortem human brain tissues. Lipids were extracted from human brain tissues and individual SL species were quantified by mass spectrometry using sphingolipidomics analysis by reverse-phase HPLC-tandem mass spectrometry. A: SL level of the NAWM from the nCNS. SL classes are presented as mean (expressed as picomoles per milligram of protein) ± SEM (n = 3). SL composition in post mortem human brain tissues derived from: NAWM from nCNS (B); Ac-MS (C); I-OND (D); In-MS (E); and NI-OND (F). The significant alterations (increase or decrease) are indicated by colored arrows. Data are shown as mean (expressed in percent of total SLs) and the pie charts were generated with GraphPad PRISM 7.01.

Compositional analysis of total SLs indicated that SM was the dominant species of the NAWM of the nCNS (**Fig. 2A**). The lipid composition of the NAWM of the nCNS was as follows: SM (72.6 ± 4.4%) followed by Cer (13.5 ± 0.09%), HexCer (11.6 ± 0.54%), LacCer (0.9 ± 0.03%), C1P (0.8 ± 0.04%), sphingoids and their derivatives (0.3 ± 0.01%), and dhCer species (0.3 ± 0.02%), respectively (Fig. 2B). The most abundant SM subspecies were identified to be: C18-SM (29.5 ± 2.6%), C24:1-SM (19.6 ± 3.1%), C16-SM (12.9 ± 2.6%), and C24-SM (11.4 ± 1.2%) (supplemental Fig. S1A). The next largest group was comprised of Cer species, which was composed of C18-Cer (52.0 ± 1.9%), C18:1-Cer (16.0 ± 2.7%) and C24:1-Cer (14.2 ± 0.2%) (supplemental Fig. S1B). Contrary to SM precursors of Cer, precursors derived from de novo Cer synthesis, i.e., dhCer species, constitute a very low amount of SLs. The major dhCer species were dhC18-Cer (39.6 ± 1.1%), dhC24:1-Cer (26.1 ± 3.0%), and dhC24:0-Cer (6.8 ± 0.9%) (supplemental Fig. S1C). Similar abundance to Cer subspecies represents HexCer subspecies that included: C18-HexCer (42.0 ± 2.6%), C24:1-HexCer (30.5 ± 0.9%), and C24:0-HexCer (7.4 ± 1.1%) (supplemental Fig. S2A). Comparatively, SLs such as LacCer subspecies: C18-LacCer (53.4 ± 3.2%); C16-LacCer (32.8 ± 4.0%); C24:1-LacCer (9.5 ± 2.4%) (supplemental Fig. S2B), as well as C1P subspecies: C18:0-C1P (40.3 ± 7.0%); C24-C1P (15.8 ± 1.7%), and C24:1-C1P (15.8 ± 1.7%) (supplemental Fig. S3C) made up a very small proportion of the entire sphingolipidome (Fig. 2A). Sphingoids and their phosphate derivatives (Fig. 2A), were also measured (supplemental Fig. S1D). The detailed molecular distribution of the individual lipid subspecies measured are summarized in supplemental Figs. S1 and S2.

Overall, whereas the lipid profile of Ac-MS lesions (Fig. 2C) seemed to be comparable with that of the NAWM of the nCNS (Fig. 2B), the lipid profile of In-MS lesions was significantly different (Fig. 2E). The most striking changes in In-MS lesions were observed for C1P (4.2-fold increase), HexCer (4.0-fold increase), Cer (4.4-fold decrease), dhCer (3.6-fold decrease), sphingoids (1.9-fold increase), and SM (1.6-fold decrease) content in comparison to Ac-MS lesions. Another observation was that the lipid profile of Ac-MS (Fig. 2C) seemed to be similar to the profile from the I-OND subgroup (Fig. 2D), whereas the profile of In-MS seemed to be comparable with that of the NI-OND controls (Fig. 2F).

### Cer and its main precursors are dependent on MS activity

**Fig. 3.**
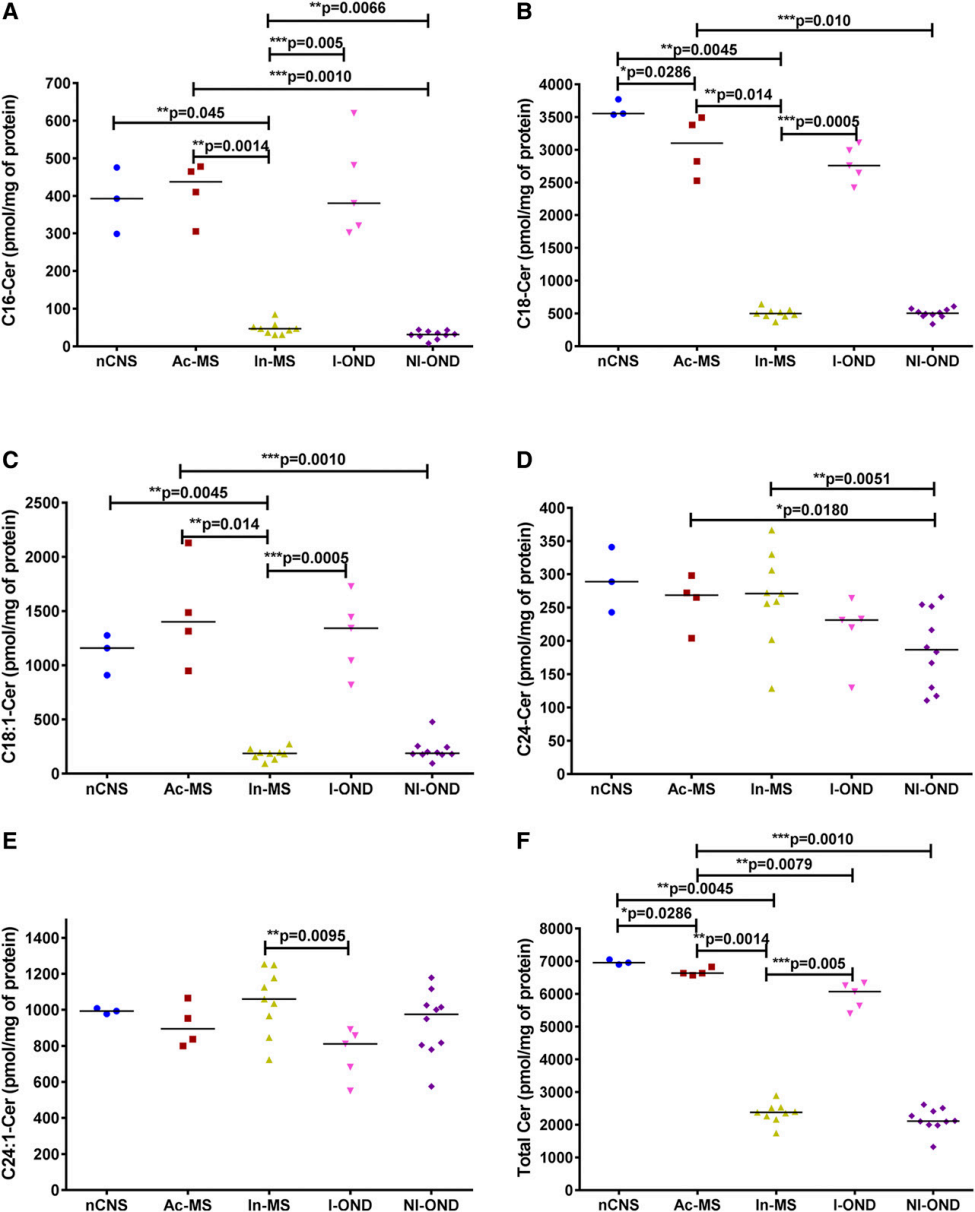
XY scatter plots of the major Cer subspecies: C16-Cer (A), C18-Cer (B), C18:1-Cer (C), C24-Cer (D), C24:1-Cer (E), and total Cer (F) in chronic MS plaques (Ac-MS and In-MS) in comparison to the NAWM of the the nCNS and ONDs (I-OND and NI-OND). The comparison between Ac-MS and In-MS subgroups was also included. The data are expressed as picomoles per milligram of protein. Horizontal bars indicate median values. Differences between groups of nonparametric data were determined by the Mann-Whitney test using GraphPad PRISM 7.01. nCNS (n = 3), Ac-MS (n = 4), In-MS (n = 9), I-OND (n = 5), NI-OND (n = 10).

To determine the relationships between the active/inactive type of MS-related chronic brain damage and bioactive lipids such as Cer and its main precursors derived from de novo Cer synthesis as well as SM hydrolysis, we determined the level of Cer, dhCer, and SM subspecies in chronic Ac- and In-MS lesions. Besides the NAWM from the nCNS, two types of reference groups, I-OND and NI-OND, were included. There was a clear decrease in total Cer level in In-MS plaques in comparison to the NAWM from the nCNS as well as the I-OND reference group (**Fig. 3F**), including major Cers of the nCNS subspecies: C16:0-Cer (Fig. 3A), C18:0-Cer (Fig. 3B), and C18:1-Cer (Fig. 3C). Contrary to In-MS plaques, in Ac-MS plaques there was only a slight decrease in Cer level in comparison to the nCNS (Fig. 3F), whereas C16:0-Cer (Fig. 3A), C18:0-Cer (Fig. 3B), and C18:1-Cer subspecies (Fig. 3C) were significantly upregulated, resulting in a significantly increased total Cer level compared with the NI-OND group (Fig. 3F). Most of the major Cer subspecies in Ac-MS lesions were also significantly increased in comparison to In-MS (Fig. 3A–C), accounting for the significant increase of the total Cer content (Figs. 2C, 3F).

**Fig. 4.**
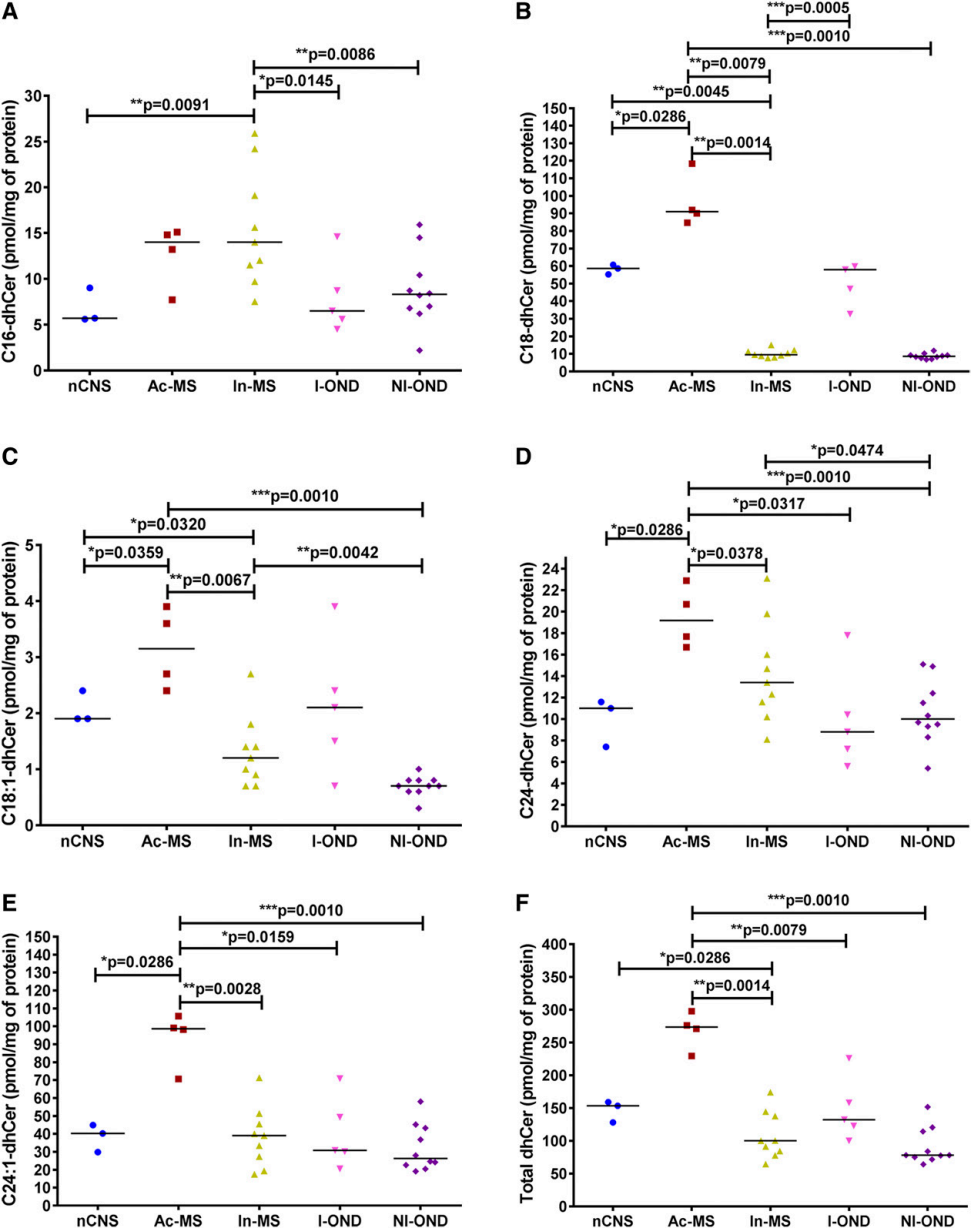
XY scatter plots of the major dhCer subspecies: C16-dhCer (A), C18-dhCer (B), C18:1-dhCer (C), C24-dhCer (D), C24:1-dhCer (E), and total dhCer (F) in chronic MS plaques (Ac-MS and In-MS) in comparison to the NAWM of the nCNS and ONDs (I-OND and NI-OND). The comparison between Ac-MS and In-MS subgroups was also included. The data are expressed as picomoles per milligram of protein. Horizontal bars indicate median values. Differences between groups of nonparametric data were determined by the Mann-Whitney test using GraphPad PRISM 7.01. nCNS (n = 3), Ac-MS (n = 4), In-MS (n = 9), I-OND (n = 5), NI-OND (n = 10).

The overall Cer level was increased in Ac-MS plaques, mostly due to their major dhCer precursors, i.e., C18:0 (**Fig. 4B**), C24:0 (Fig. 4D), and C24:1 (Fig. 4E), indicating that de novo Cer synthesis was active (Fig. 4F). Analyses of dhCer species in In-MS lesions compared with the nCNS and the I-OND group elicited no statistically significant differences on major dhCer types except for a significant decrease of C18:0-dhCer (Fig. 4B), accounting for the significantly decreased total dhCer level (Fig. 4F).

**Fig. 5.**
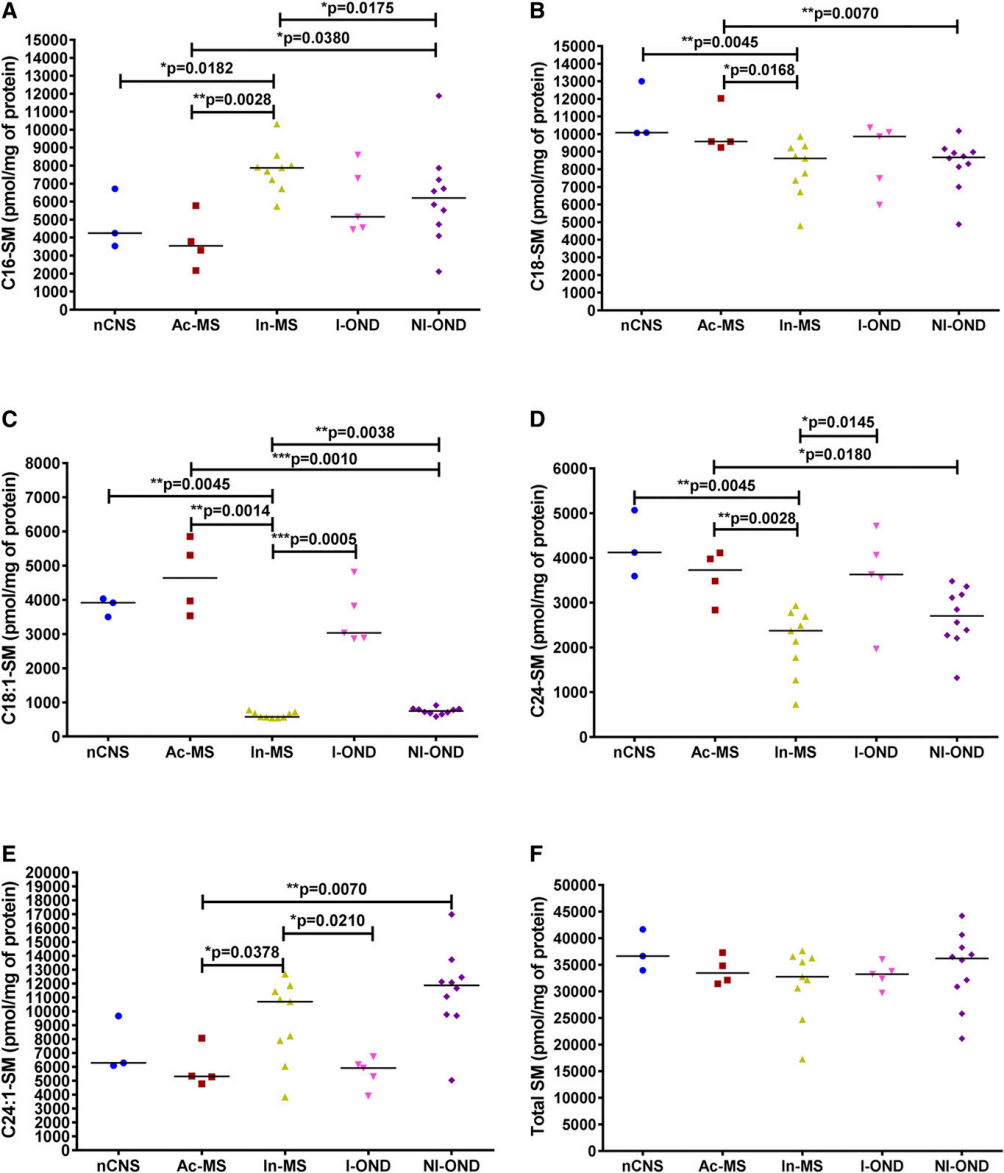
XY scatter plots of the major SM subspecies: C16-SM (A), C18-SM (B), C18:1-SM (C), C24-SM (D), C24:1-SM (E), and total SM (F) in chronic MS plaques (Ac-MS and In-MS) in comparison to the NAWM of the the nCNS and ONDs (I-OND and NI-OND). The comparison between Ac-MS and In-MS subgroups was also included. The data are expressed as picomoles per milligram of protein. Horizontal bars indicate median values. Differences between groups of nonparametric data were determined by the Mann-Whitney test using GraphPad PRISM 7.01. nCNS (n = 3), Ac-MS (n = 4), In-MS (n = 9), I-OND (n = 5), NI-OND (n = 10).

As shown in **Fig. 5**, analyses of SM subspecies in In-MS lesions indicated a significant decrease of C18:0-SM (Fig. 5B), C18:1-SM (Fig. 5C), and C24-SM (Fig. 5D) subspecies compared with the nCNS, suggesting that the SM→Cer pathway is active. Contrary to In-MS plaques in Ac-MS lesions, no significant differences regarding major SM subspecies were observed (Fig. 5).

### Glycosylated Cer derivative level is involved in MS activity

**Fig. 6.**
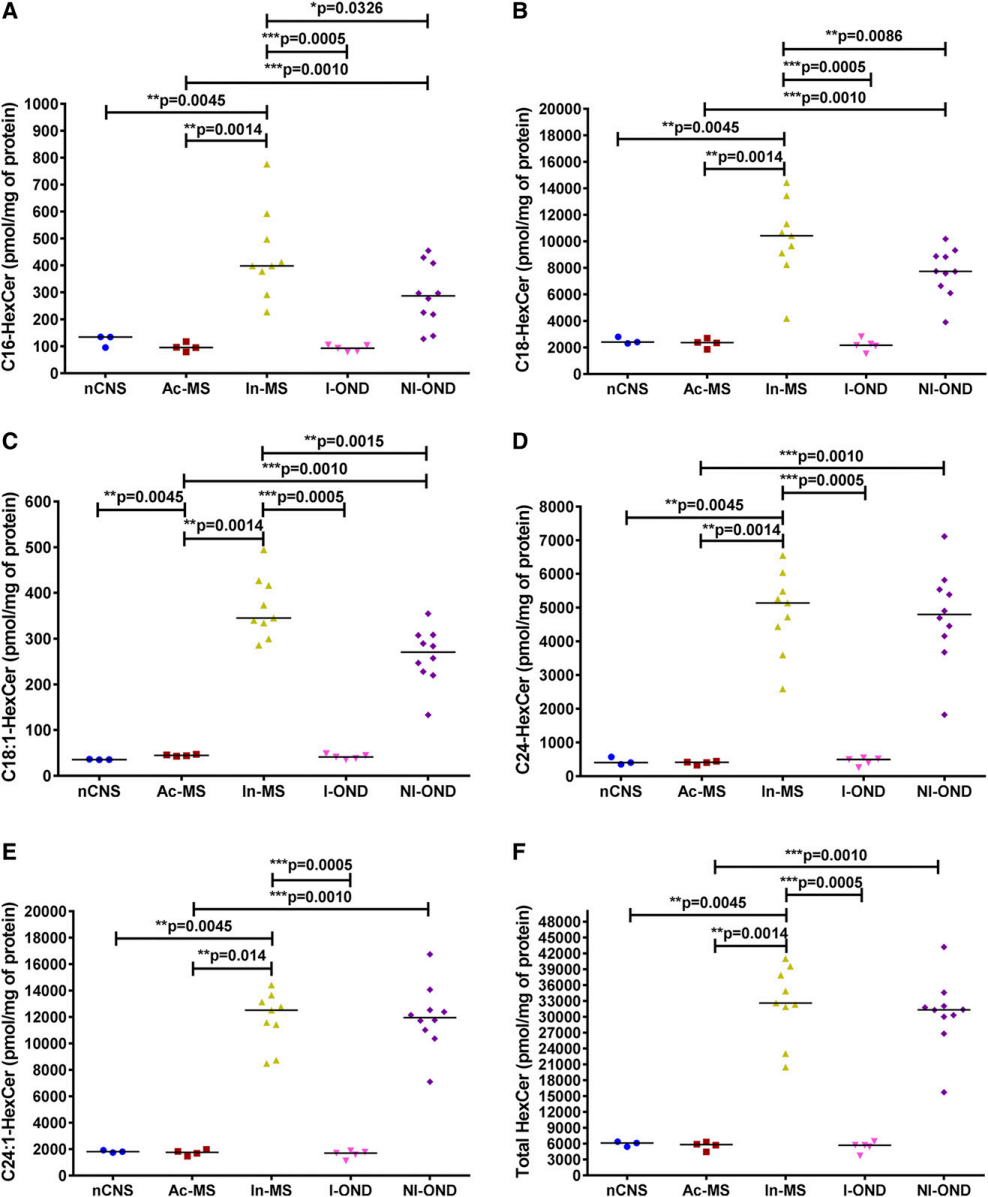
XY scatter plots of the major HexCer subspecies: C16-HexCer (A), C18-HexCer (B), C18:1-HexCer (C), C24-HexCer (D), C24:1-HexCer (E), and total HexCer (F) in chronic MS plaques (Ac-MS and In-MS) in comparison to the NAWM of the nCNS and ONDs (I-OND and NI-OND). The comparison between Ac-MS and In-MS subgroups was also included. The data are expressed as picomoles per milligram of protein. Horizontal bars indicate median values. Differences between groups of nonparametric data were determined by the Mann-Whitney test using GraphPad PRISM 7.01. nCNS (n = 3), Ac-MS (n = 4), In-MS (n = 9), I-OND (n = 5), NI-OND (n = 10).

It has recently been reported that glycosphingolipids may participate in CNS chronic inflammation (17), and their levels in CSF may reflect disease progression (28). Therefore, we aimed to determine the glycosylated Cer level, i.e., HexCer and LacCer profile, in post mortem tissues in chronic MS lesions with or without features of activity (**Figs. 6**, ** 7**). First, we analyzed whether HexCer species were modified according to disease activity. In Ac-MS plaques, there were no significant HexCer level changes compared with the nCNS, whereas C16-HexCer (Fig. 6A), C18-HexCer (Fig. 6B), C18:1-HexCer (Fig. 6C), C24-HexCer (Fig. 6D), and C24:1-HexCer (Fig. 6E) were significantly downregulated compared with the NI-OND group. In In-MS lesions, all HexCer subspecies [C16-HexCer (Fig. 6A), C18-HexCer (Fig. 6B), C18:1-HexCer (Fig. 6C), C24-HexCer (Fig. 6D), and C24:1-HexCer (Fig. 6E)] were significantly upregulated in comparison to the nCNS (Fig. 2B) and Ac-MS lesions (Fig. 2C) as well as the I-OND control (Fig. 2D), accounting for a 4.0-fold increase of total HexCer (Figs. 2E, 6F).

**Fig. 7.**
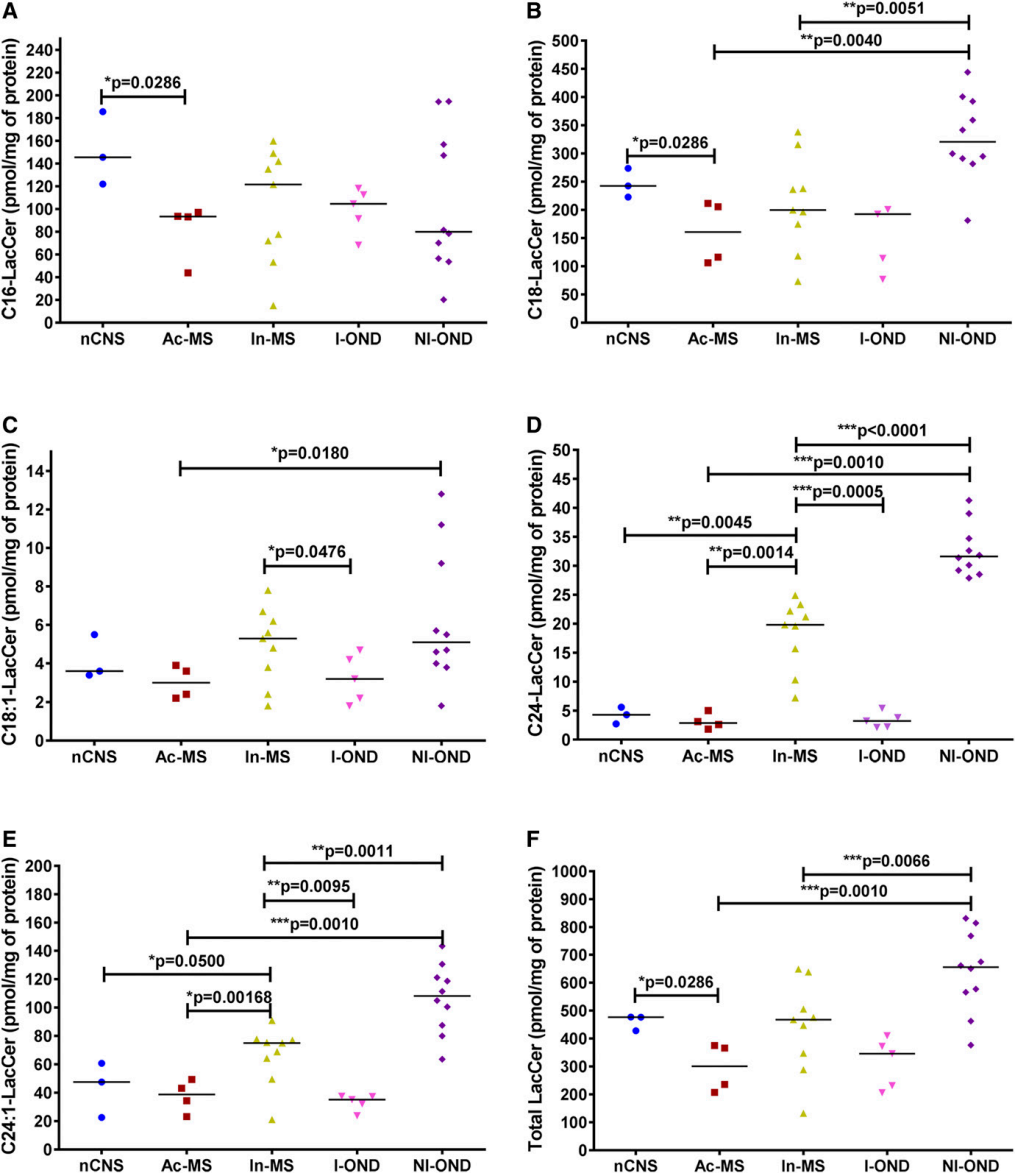
XY scatter plots of the major LacCer subspecies: C16-LacCer (A), C18-LacCer (B), C18:1-LacCer (C), C24-LacCer (D), C24:1-LacCer (E), and total LacCer (F) in chronic MS plaques (Ac-MS and In-MS) in comparison to the NAWM of the nCNS and ONDs (I-OND and NI-OND). The comparison between Ac-MS and In-MS subgroups was also included. The data are expressed as picomoles per milligram of protein. Horizontal bars indicate median values. Differences between groups of nonparametric data were determined by the Mann-Whitney test using GraphPad PRISM 7.01. nCNS (n = 3), Ac-MS (n = 4), In-MS (n = 9), I-OND (n = 5), NI-OND (n = 10).

Next, we investigated whether the MS activity dependent on altered HexCer level (Fig. 6) in the studied plaques had any influence on the level of LacCer (Fig. 7). Less clear discriminative changes in the LacCer level between different types of MS lesions were observed. Contrary to our expectation in chronic MS plaques, there were no significant LacCer level changes compared with the nCNS as well as the OND groups. Indeed, the C16-LacCer level in In-MS plaques was significantly decreased in comparison to the nCNS (Fig. 7A), whereas the C24-LacCer level in In-MS plaques was significantly increased in comparison to the nCNS, Ac-MS lesions, and the I-OND group (Fig. 7D).

Interestingly, some LacCer species, i.e., C24-LacCer (Fig. 7D) and C24:1-LacCer (Fig. 7E), were significantly decreased in Ac- and In-MS lesions compared with the NI-OND controls.

### C1P level reflects disease progression

**Fig. 8.**
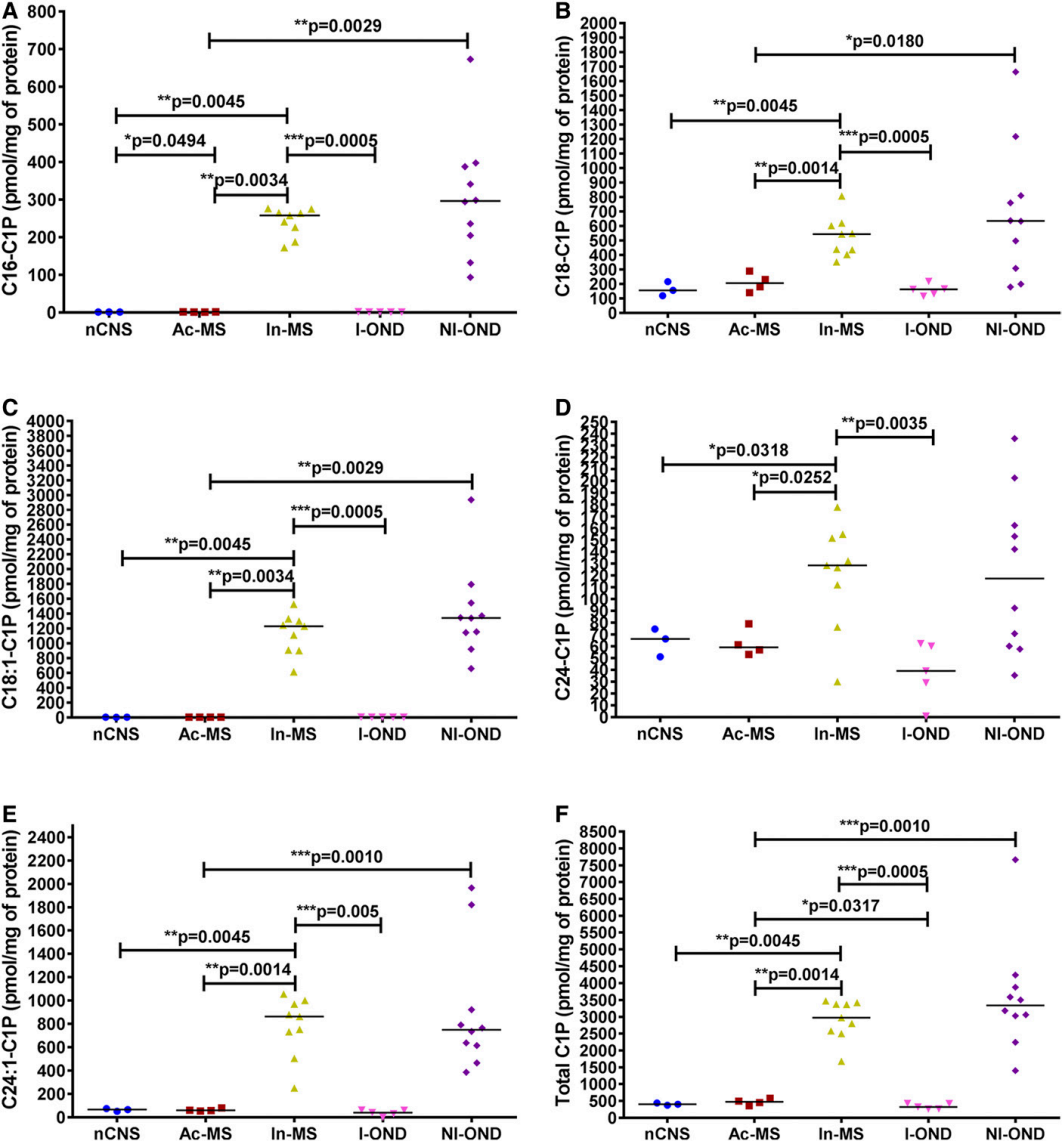
XY scatter plots of the major C1P subspecies: C16-C1P (A), C18-C1P (B), C18:1-C1P (C), C24-C1P (D), C24:1-C1P (E), and total C1P (F) in chronic MS plaques (Ac-MS and In-MS) in comparison to the NAWM of the nCNS and ONDs (I-OND and NI-OND). The comparison between Ac-MS and In-MS subgroups was also included. The data are expressed as picomoles per milligram of protein. Horizontal bars indicate median values. Differences between groups of nonparametric data were determined by the Mann-Whitney test using GraphPad PRISM 7.01. nCNS (n = 3), Ac-MS (n = 4), In-MS (n = 9), I-OND (n = 5), NI-OND (n = 10).

We next assessed whether C1P subtypes are involved in disease activity (**Fig. 8**). Surprisingly, an enormous upregulation of C16-C1P (Fig. 8A), C18-C1P (Fig. 8B), C18:1-C1P (Fig. 8C), and C24:1-C1P (Fig. 8D) subspecies was observed in In-MS lesions compared with the nCNS, Ac-MS lesions, and the I-OND group, as reflected by a striking increase of total C1P (Fig. 8F). Contrary to that, Ac-MS plaques indicated a significant decrease of C1P subspecies in comparison to the NI-OND group (Fig. 8A–F).

### Sphingoid alterations in progressive MS

**Fig. 9.**
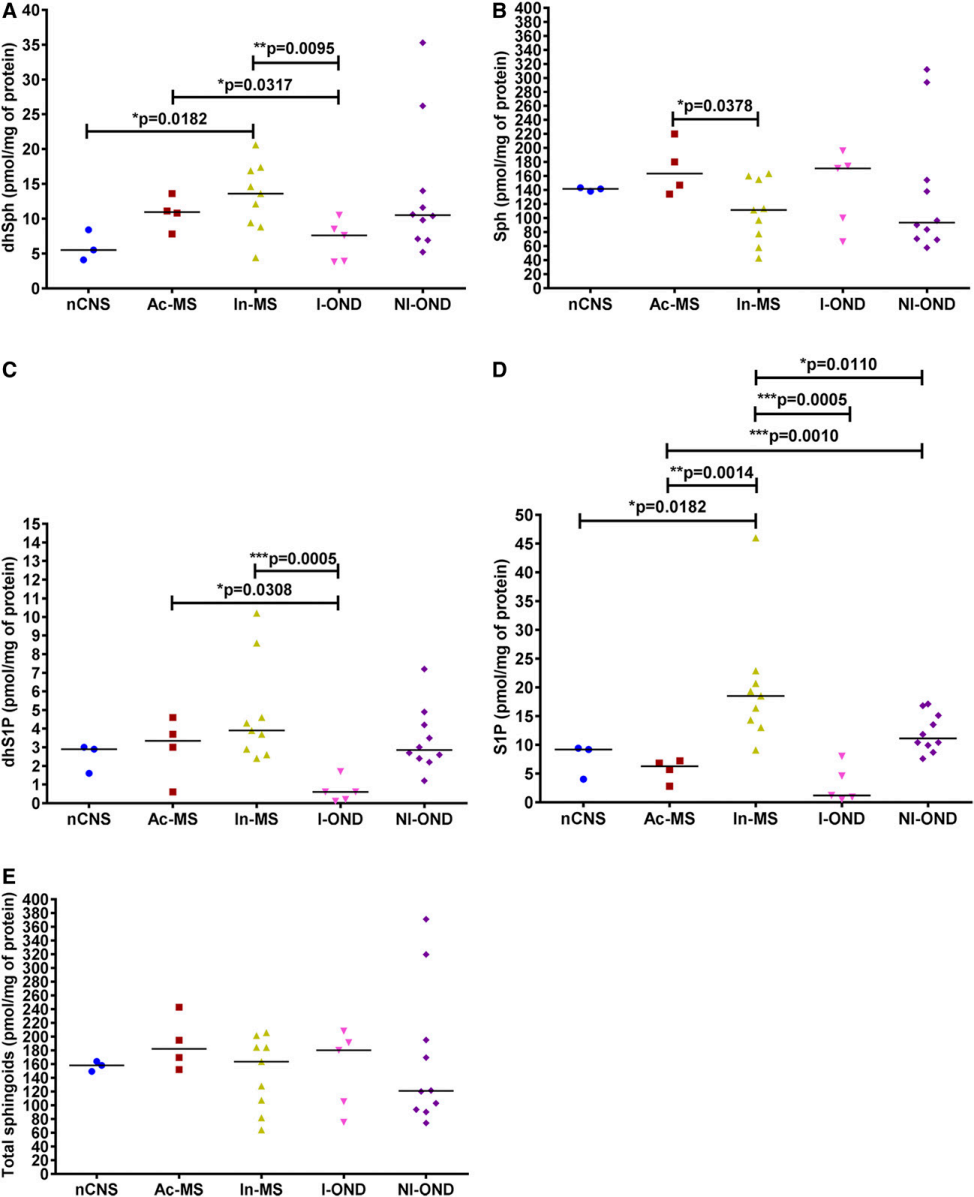
XY scatter plots of the major sphingoid subspecies: dhSph (A), Sph (B), dhS1P (C), S1P (D), and total sphingoids (E) in chronic MS plaques (Ac-MS and In-MS) in comparison to the NAWM of the nCNS and ONDs (I-OND and NI-OND). The comparison between Ac-MS and In-MS subgroups was also included. The data are expressed as picomoles per milligram of protein. Horizontal bars indicate median values. Differences between groups of nonparametric data were determined by the Mann-Whitney test using GraphPad PRISM 7.01. nCNS (n = 3), Ac-MS (n = 4), In-MS (n = 9), I-OND (n = 5), NI-OND (n = 10).

To further understand the mechanism of MS course, sphingoids and their derivatives (dhSph and Sph as well as dhS1P and S1P, respectively) were quantified in particular phases of the disease (**Fig. 9**). Sph content was increased in Ac-MS lesions in comparison to In-MS (Fig. 9B). Elevation in S1P occurred in In-MS plaques compared with the nCNS, I-OND, and NI-OND groups as well as Ac-MS (Fig. 9D), while its precursor, dhS1P, showed significant alteration only compared with the I-OND group (Fig. 9C).

## DISCUSSION

SLs belong to one of the several families of bioactive lipids that activate specific G protein-coupled receptors, thereby acting in both stages involved in MS background: inflammation (34) and neurodegeneration (35). Each phase requires the concerted action of such SL mediators, which are likely to interact and engage in the pathophysiological cross-talk. As an important component of the CNS, SLs could affect the viability of brain cells (oligodendrocytes, neurons, and astrocytes), which is mediated by their signaling. Recent studies indicated that Cer (18–21, 26) and its glycosylated derivatives (17, 28) have attracted the most attention in the MS field. Because SL pathway changes have recently emerged as key factors in CNS disorders, including MS, we investigated aberrant SL metabolism in MS post mortem tissues, which might be dependent on the disease progression. The regulation of a vastly intertwined network of bioactive SL molecules with their extensive structural diversity is complex and still not unraveled with respect to MS. Consequently, the full elucidation of their role in the different phases of the disease pathogenic mechanisms, from acute inflammation and its resolution to chronic inflammation with parallel neurodegeneration, represents conceivably one of the biggest challenges.

Our data suggest different pathological scenarios for Ac- and In-MS-related damage differentiated mainly by a Cer source. Cer is the central hub of the SL pathway, which includes dhCer, SM, HexCer (gluco- and galactosylceramides), LacCer and sphingoid bases (Sph and dhSph) and sphingoid base 1-phosphates (dhS1P and S1P), and other SLs. In order to test the association between the features of chronic MS-related brain damage and SL levels, we applied sphingolipidomics to quantify bioactive SL mediators in post mortem tissues obtained from subjects with advanced stages of MS. There are two main pathways of Cer production: de novo biosynthesis and endocytic recycling. Which of these pathways dominates for supplying Cer depends on the cell type and specific conditions and remains to be elucidated. Although it was originally thought that SMase was the key enzyme responsible for Cer generation, our ex vivo studies implicate de novo SL biosynthesis, as indicated by elevated levels of C18:0-dhCer (Fig. 4B), C24:0-dhCer (Fig. 4D), and C24:1-dhCer (Fig. 4E), in the Ac-MS lesions. The de novo pathway for Cer generation via serine palmitoyltransferase (SPT) activation has already been reported in experimental autoimmune encephalomyelitis (26). In line with this, the use of C16-Cer and/or Cer synthase, specifically CerS6 (36) as well as C16-dhCer (37), as biomarkers of MS early activity and/or its progression has been elaborated. On the contrary, we have found that, in In-MS lesions, Cer may be derived from SM hydrolysis. We did observe a significant decrease of three SM subtypes [C18:0-SM (Fig. 5B), C18:1-SM (Fig. 5C), and C24:0-SM (Fig. 5D)] in In-MS plaques in comparison to normal brain as well as Ac-MS lesions. So far, the increased level of acid SMase activity and the increased number of exosomes that carry acid SMase have been reported in the CSF of MS patients (compared with those with other CNS diseases) (38). Interestingly, acid SMase activity did not differ significantly between the sera from patients with relapsing-remitting, secondary-progressive, and primary-progressive MS, and no association was found between acid SMase activity and the clinical or radiological signs of the disease activity (39). These data suggest that SLs in CSF might be more relevant as MS biomarkers than in serum. Interestingly, although Cer is generated intracellularly, it can also be found in biological fluids, i.e., plasma (21), where it binds to microvesicles such as exosomes (20). It is also of interest that exogenous Cer was shown to induce acid SMase activity or stimulate the de novo pathway to produce more intracellular Cer, pointing to the existence of a Cer-triggered paracrine amplification loop to increase Cer levels in cells (40).

Cer is the structural backbone of SLs and a precursor of complex SLs. Consequently, further perturbation in its metabolism may have an important implication for disease progression. First, our data indicate that Cer could be metabolized to its glycosylated derivatives, as suggested by the significant increase of all HexCer species examined, e.g., C16:0-HexCer (Fig. 6A), C18:0-HexCer (Fig. 6B), C18:1-HexCer (Fig. 6C), C24:0-HexCer (Fig. 6D), and C24:1-HexCer (Fig. 6E), in In-MS lesions compared with both normal brain and Ac-MS plaques. Interestingly, several SL species were already found to be elevated in the CSF of MS compared with controls, including C16:0-Cer, C24:0-Cer, and C16:0-HexCer, indicating that the SM→Cer→HexCer pathway in MS might be relevant to the effect of damage to neurons (19). It should be emphasized that correlations between C16:0-HexCer and C24:1-HexCer in CSF and the degree of disability in the Expanded Disability Status Scale were previously observed (28), which further supports the concept of CSF SL components as relevant MS biomarkers. The observed increase of all HexCer subspecies (Fig. 6) could potentially reflect and implicate further alterations in more complex glycosylated SLs in the progressive phase of MS. Contrary to our expectations, we did not observe discriminative differences with respect to the LacCer subspecies level (Fig. 7). However, the increased level of LacCer was already observed in MS brain tissues (17). Glycosylated Cers, specifically C24:1-LacCer and C16-GluCer, have also been proposed as lipid-based biomarkers for MS (37). The discrepancy between these studies and our findings might result from the heterogeneous pathology of the MS-affected brain tissue specimens subjected to analysis. Moreover, in the previous studies of CSF findings, histopathological examination was not conducted and/or the MS clinical subtype was not specified.

Second, we found some C1P subspecies, specifically C16:0-C1P (Fig. 8A), C18:0-C1P (Fig. 8B), C18:1-C1P (Fig. 8C), C24:0-C1P (Fig. 8D), and C24:1-C1P (Fig. 8E), to be increased, resulting in 5.3-fold increase of total C1P (Fig. 8E) during the progressive MS course. C1P in MS is most likely generated by Cer kinase action, and this Ca^2+^ ion-dependent enzyme has been reported to be highly active in brain tissue (41), although alternative pathways cannot be completely excluded. For example, the transfer of fatty acyl chain to S1P or the degradation of SM by the D-type phospholipases would render C1P directly (42). Major sources for C1P are macrophages and leaky damaged cells (43).

The striking increase of C1P, as reported here for the first time in chronic/progressive MS, undoubtedly has biological meaning and diagnostic value. It is of note that most of the pro-inflammatory activities of Cer seem to be mediated through C1P rather than its S1P metabolites (44). For example, C1P and S1P could act as chemoattractants for tumor cells, and their increased level in several organs after radio-/chemotherapy indicates induction of an unwanted pro-metastatic environment as a side effect of oncologic treatment (45); thus, their chemotactic gradient became a legitimate target for anti-metastatic therapies (46). Other activities of C1P include its capability to mediate arachidonic acid release (47) and also to activate group IVA cytosolic phospholipase A_2_α (cPLA_2_α), which is the rate-limiting releaser of arachidonic acid used for production of pro-inflammatory eicosanoids (48). Furthermore, it has been proposed that C1P transfer protein prevents excess C1P accumulation after its production by CERK in the *trans*-Golgi network, thereby regulating cPLA_2_α action and diminishing arachidonic acid release and downstream generation by eicosanoid producers such as COX-1 or COX-2 (49). These observations suggested that targeting C1P level at the *trans*-Golgi network potentially targets cPLA_2_α-mediated eicosanoid biosynthesis and the pro-inflammatory pathological process. Interestingly, C1P specifically increases the transport of P-glycoprotein, an ATP-driven efflux pump that regulates the permeability of the blood-brain barrier via COX-2/prostaglandin E2 signaling, which offers clinical benefits for drug delivery into the CNS to modulate neuroprotection (50).

Although the pro-inflammatory properties of intracellular C1P are well established, as discussed above, increasing experimental evidence indicates that C1P can also exert anti-inflammatory actions in some particular cell types or tissues. Many of the anti-inflammatory effects of C1P include blockade or counteraction of Cer-induced inflammatory responses. In line with this connection, one of the initial anti-inflammatory actions of C1P might be inhibition of stimulated Cer production, which was reported to occur in macrophages through blockade of SPT (51), acid SMase (52) activities; the effects associated to the anti-apoptotic effect of C1P. In addition, C1P was shown to be a potent inhibitor of TNF-α-converting enzyme (53), thereby emphasizing its anti-inflammatory action.

Interestingly, it has also been reported that C1P promotes macrophage chemoattractant protein-1 (MCP-1) released in different types of cells, and this chemokine was revealed to be a major mediator of C1P-stimulated cell migration events (54, 55). However, C1P-stimulated macrophage migration could be blocked by PA, a glycerophospholipid structurally related to C1P (56).

In conclusion, our investigation of chronic MS lesions in the brain revealed different SL molecules supposed to differentiate inflammatory and neurodegenerative processes underlying MS pathology. These guardian SL molecules and their corresponding pathologic pathways could be potentially exploited in both Ac-MS and In-MS forms. More neuropathological research is needed in order to define the relationship between the accumulation of these particular SLs and MS activity or progression. All of these SL molecules might serve as relevant biomarkers and, hopefully, also platforms for novel therapies.

### Data availability

The data supporting this study are available in the article and are available from the corresponding author upon reasonable request.

## Supplementary Material

Supplemental Data
