# Supplementary material for: Distinctive sphingolipid patterns in chronic multiple sclerosis lesions
Source: J Lipid Res. 2020 Aug 7;61(11):1464–79. doi: 10.1194/jlr.RA120001022 (PMC7604719; doi:10.1194/jlr.RA120001022)
Supplement: Supplemental Data [file supp_61_11_1464__index.html]

Distinctive sphingolipid patterns in chronic multiple sclerosis lesions — Potential sphingolipid biomarkers in progressive MS — Distinctive sphingolipid patterns in chronic multiple sclerosis lesions — Supplemental Data 

# Distinctive sphingolipid patterns in chronic multiple sclerosis lesions

## Supplemental Data

- Supplemental Data - 2 Supplemental Figures
