# Supplementary material for: Distinctive sphingolipid patterns in chronic multiple sclerosis lesions
Source: J Lipid Res. 2020 Aug 7;61(11):1464–79. doi: 10.1194/jlr.RA120001022 (PMC7604719; doi:10.1194/jlr.RA120001022)
Supplement: Supplemental Data [file supp_RA120001022_162042_1_supp_577422_q1ngpp.pdf]

Supplemental Material

Distinctive sphingolipid patterns in chronic multiple sclerosis lesions

Maria Podbielska[1,6], Zdzislaw M. Szulc[1], Toshio Ariga[5], Anna Pokryszko-Dragan[3], Wojciech Fortuna[4,7], Małgorzata Bilinska[3], Ryszard Podemski[3], Ewa Jaskiewicz[8], Ewa Kurowska[6], Robert K. Yu[5], Edward L. Hogan[2,†]

From [1]Department of Biochemistry & Molecular Biology and [2]Department of Neurology, Medical University of South Carolina, Charleston, SC 29425, USA; [†]Deceased

[3]Department of Neurology and [4]Department of Neurosurgery, Wroclaw Medical University, 50-556 Wroclaw, Poland;

[5]Department of Neuroscience and Regenerative Medicine, Augusta University, Medical of College of Georgia, Augusta, GA 30912, USA;

[6]Laboratory of Microbiome Immunobiology and [7]Bacteriophage Laboratory and [8]Laboratory of Glycobiology, Ludwik Hirszfeld Institute of Immunology & Experimental Therapy, Polish Academy of Sciences, 53-114 Wroclaw, Poland

Running tittle: Potential sphingolipid biomarkers in progressive MS

To whom correspondence should be addressed: Maria Podbielska, Ludwik Hirszfeld Institute of Immunology & Experimental Therapy, Polish Academy of Sciences, Laboratory of Microbiome Immunobiology, Rudolfa Weigla 12, 53-114, Wroclaw, Poland; Tel: +48-71-370-99-12, Fax: +48-71-337-21-71, E-mail: maria.podbielska@hirszfeld.pl

The abbreviations used are: C1P, ceramide 1-phosphate; Cer, ceramide; cPLA$_2\alpha$, cytosolic phospholipase A$_2\alpha$; CSF, cerebrospinal fluid; dhCer, dihydroceramide; EAE, experimental autoimmune encephalomyelitis; GluCer, glucosylceramide; H&E, hematoxylin and eosin; HexCer, hexosylceramide; HPLC-MS/MS, high-performance liquid chromatography-tandem mass spectrometry; ISs, internal standards; LacCer, lactosylceramide; LFB, Luxol fast blue; MS, multiple

sclerosis; Ac-MS, chronic active multiple sclerosis; In-MS, chronic inactive multiple sclerosis; NAWM, normal appearing white matter; nCNS, normal central nervous system; OND, other neurological diseases; I-OND, inflammatory other neurological diseases; NI-OND, non-inflammatory other neurological diseases; SL, sphingolipid; SM, sphingomyelin; Sph, sphingosine; SPT, serine palmitoyltransferase.

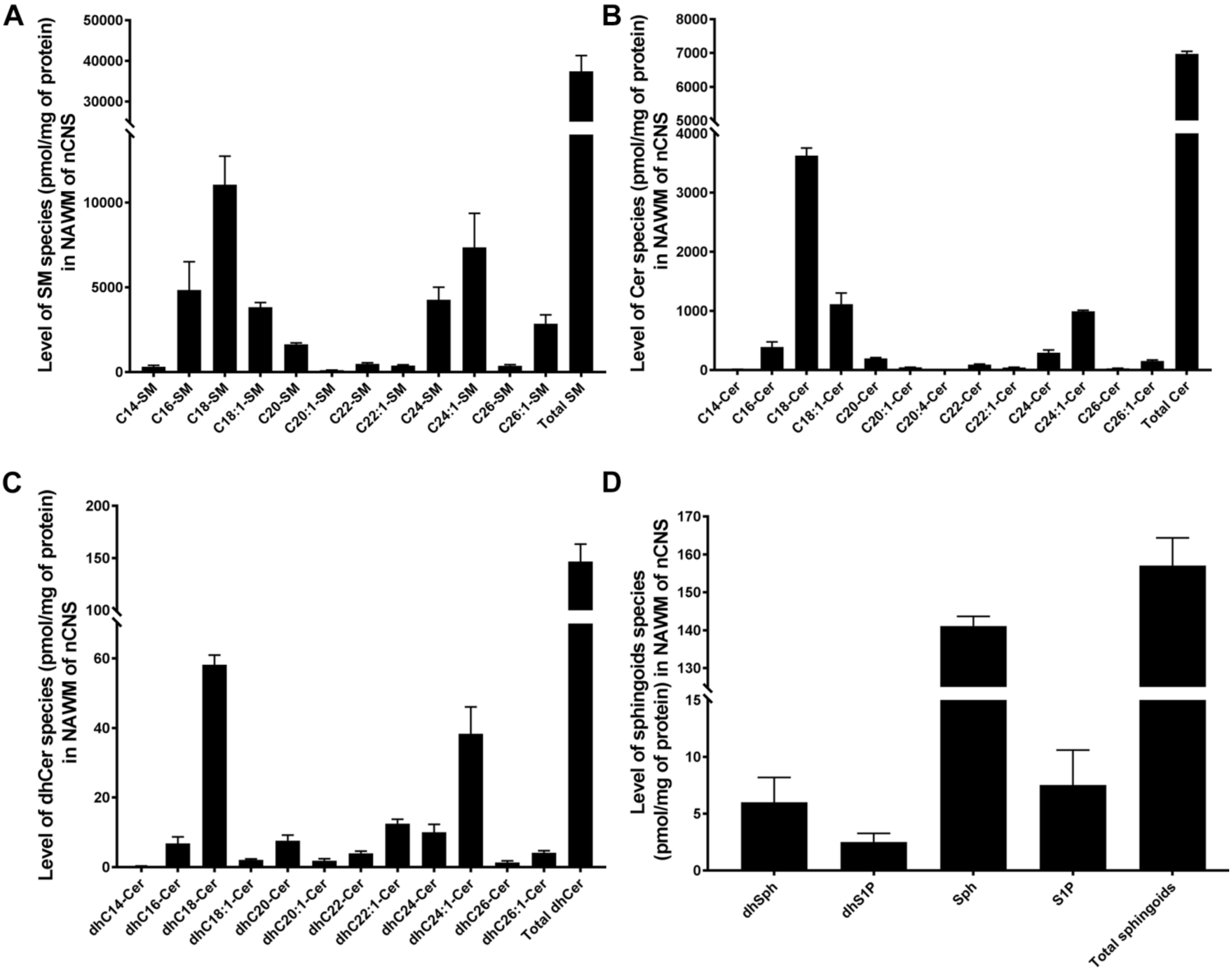

Supplemental Figure S1. SL profile of NAWM from nCNS. Lipids were extracted with organic solvent and the levels of **A**). SM, **B**). Cer, **C**). dhCer and **D**). sphingoids were quantified by mass spectrometry using sphingolipidomics analysis by reverse phase HPLC-MS/MS. All species were identified by their specific parent-daughter ion mass transition and retention time. The above SL subclasses are mean (expressed as pmol/mg of protein) ± SEM. n=3; dhCer, dihydroceramide; dhS1P, dihydrosphingosine-1-phosphate; dhSph, dihydrosphingosine; NAWM, normal appearing white matter; nCNS, normal CNS; S1P, sphingosine-1-phosphate Sph, sphingosine.

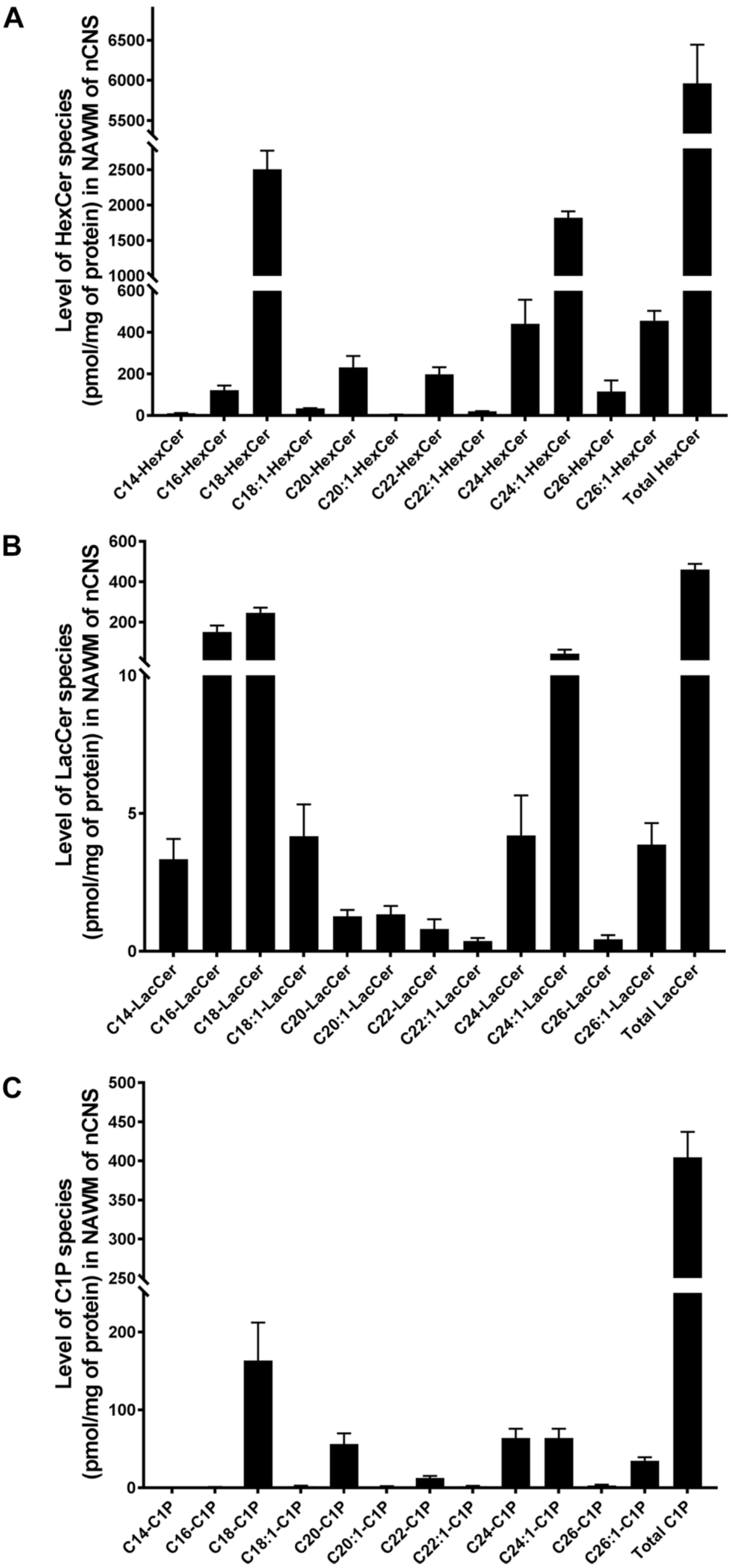
A
Level of HexCer species
(pmol/mg of protein) in NAWM of nCNS
6500
6000
5500
2500
2000
1500
1000
600
400
200
0
C14-HexCer
C16-HexCer
C18-HexCer
C18:1-HexCer
C20-HexCer
C20:1-HexCer
C22-HexCer
C22:1-HexCer
C24-HexCer
C24:1-HexCer
C26-HexCer
C26:1-HexCer
Total HexCer
B
Level of LacCer species
(pmol/mg of protein) in NAWM of nCNS
600
400
200
10
5
0
C14-LacCer
C16-LacCer
C18-LacCer
C18:1-LacCer
C20-LacCer
C20:1-LacCer
C22-LacCer
C22:1-LacCer
C24-LacCer
C24:1-LacCer
C26-LacCer
C26:1-LacCer
Total LacCer
C
Level of C1P species
(pmol/mg of protein) in NAWM of nCNS
500
450
400
350
300
250
200
100
0
C14-C1P
C16-C1P
C18-C1P
C18:1-C1P
C20-C1P
C20:1-C1P
C22-C1P
C22:1-C1P
C24-C1P
C24:1-C1P
C26-C1P
C26:1-C1P
Total C1P

Supplemental Figure S2. Profile of complex SLs of NAWM from nCNS. Lipids were extracted with organic solvent and the levels of **A**). HexCer, **B**). LacCer and **C**). C1P were quantified by mass spectrometry using sphingolipidomics analysis by reverse phase HPLC-MS/MS. All species were identified by their specific parent-daughter ion mass transition and retention time. The above SL subclasses are mean (expressed as pmol/mg of protein) ± SEM. n=3; C1P, ceramide-1-phosphate; HexCer, hexosylceramide; LacCer, lactosylceramide; NAWM, normal appearing white matter; nCNS, normal CNS.
